# Reformatting Rituximab into Human IgG2 and IgG4 Isotypes Dramatically Improves Apoptosis Induction *In Vitro*

**DOI:** 10.1371/journal.pone.0145633

**Published:** 2015-12-29

**Authors:** Jennifer D. Könitzer, Annette Sieron, Angelika Wacker, Barbara Enenkel

**Affiliations:** 1 Boehringer Ingelheim, Division Research Germany, Immune Modulation and Biotherapeutics Discovery, Biberach/Riß, Germany; 2 Boehringer Ingelheim, Bioprocess and Pharmaceutical Development Germany, Biberach/Riß, Germany; 3 Boehringer Ingelheim, Biopharma Operations Germany, Biberach/Riß, Germany; Chang Gung University, TAIWAN

## Abstract

The direct induction of cell death, or apoptosis, in target cells is one of the effector mechanisms for the anti CD20 antibody Rituximab. Here we provide evidence that Rituximab’s apoptotic ability is linked to the antibody IgG isotype. Reformatting Rituximab from the standard human IgG1 heavy chain into IgG2 or IgG4 boosted *in vitro* apoptosis induction in the Burkitt’s lymphoma B cell line Ramos five and four-fold respectively. The determinants for this behavior are located in the hinge region and CH1 domain of the heavy chain. By transplanting individual IgG2 or IgG4 specific amino acid residues onto otherwise IgG1 like backbones, thereby creating hybrid antibodies, the same enhancement of apoptosis induction could be achieved. The cysteines at position 131 of the CH1 domain and 219 in the hinge region, involved in IgG2 and IgG4 disulfide formation, were found to be of particular structural importance. Our data indicates that the hybrid antibodies possess a different CD20 binding mode than standard Rituximab, which appears to be key in enhancing apoptotic ability. The presented work opens up an interesting engineering route for enhancing the direct cytotoxic ability of therapeutic antibodies.

## Introduction

The chimeric CD20 antibody Rituximab (RTX) combines a murine variable region with human constant domains and has been approved for the treatment of B cell malignancies since 1997. RTX’s efficacy has been associated with a number of mechanisms; including the activation of immune effector functions such as complement dependent lysis (CDC) or antibody-dependent cell mediated cytotoxicity (ADCC) as well as the direct induction of apoptosis in CD20 expressing tumor cells [1–5]. Therapeutic antibodies in general are able to elicit apoptosis in target cells by activating death receptors, inhibiting growth or survival pathways by blocking receptor-ligand interactions or crosslinking cell surface antigens which induces pro-apoptotic signaling [6]. The exact apoptotic mechanisms of anti CD20 mAb are still being debated. It has been reported that CD20 crosslinking on the cell surface [7,8] leads to the activation of tyrosine kinases [9–12] and an increase in intracellular calcium levels [8,11,13]. While some works have found classic hallmarks of apoptosis such as caspase activation [13,14], other authors describe cell death to be independent of caspase and mitochondrial pathways [11,15]. The cell death process has been described to involve intracellular actin polymerization and lysosome rupture [16,17] as well as the release of reactive oxygen species [18].

The development of therapeutic antibodies has thus far largely focused on the IgG class and its four subtypes. Of those, only IgG1, IgG2 and IgG4 have been utilized due to IgG3’s extensive allotypic variance in the constant domain and proneness to proteolytic cleavage caused by its long hinge region [19,20]. The human IgG subclasses differ with regards to their biological role *in vivo*. IgG1, the most abundant IgG comprising 60% of all serum IgG, is largely formed in response to protein antigens. IgG2 (25%) recognizes carbohydrate antigens associated with bacterial infection whereas IgG4 (5%) occurs in response to persistent antigen stimulation and is thus considered to possess anti-inflammatory propertied [20,21]. The IgG subtypes exhibit stark differences in engaging host effector functions due to their varying affinities to complement proteins and Fcγ receptors present on immune cells. IgG1 is considered an active isotype, with the ability to interact with C1, FcγRI, FcγRII and FcγRIII, and thus being able to elicit both ADCC and CDC [20,22]. IgG2 only interacts with FcγRII bearing leukocytes but lacks the ability to induce ADCC and CDC and has therefore been used in therapeutic antibodies where the recruitment of host effector functions would be detrimental to the therapeutic concept [19,21]. IgG4 has also been applied as an inactive isotype. Some authors, however, have shown that IgG4 is able to recruit Fcγ receptors and has the capacity to induce ADCC under certain conditions [23,24].

While multiple studies have been undertaken to identify and mutate the constant domain residues responsible for controlling ADCC and CDC, with both effector enhancing and attenuating mutations having been described [25–29], the relationship between the IgG subclasses and apoptosis induction has thus far not been investigated. Here we report that, using Rituximab as a model antibody, IgG2 and IgG4 subtypes are more effective at eliciting apoptosis in CD20 expressing target cells *in vitro* than IgG1. We quantified cell death induction by measuring the externalization of the cell membrane lipid phosphatidylserine as a marker for apoptosis [30]. We found that IgG2 and IgG4’s increased apoptotic ability is influenced by the hinge and CH1 domains of the heavy chain and that transplanting hallmark amino acid residues onto IgG1, thereby creating hybrid antibodies, increases IgG1’s cytotoxic potential. The hybrid antibodies were found to possess a distinct CD20 binding mode and modulated ADCC activity in comparison to the IgG1 control. We propose this approach as an interesting engineering design option in enhancing the direct cytotoxic efficacy of therapeutic antibodies; particularly in indications such as oncology where the directed ablation of target expressing cells is an essential pharmacological mode of action.

## Materials and Methods

### Expression vector construction

Expression vectors for the heavy and light chain of RTX were constructed by inserting the coding sequences into separate pTT5 [31] vectors using routine molecular biology techniques [32]. The point mutations required to obtain amino acid exchanges in the heavy chain of the hybrid antibodies were introduced by whole plasmid site specific mutagenesis [33]. Mutagenic primers (30-45bp) were ordered from MWG Biotech. The Phusion Hot Start Polymerase (NEB) was employed for amplification. In a total reaction volume of 25μl, 50ng of the parental heavy chain plasmid was mixed with 0.3μM of each primer, 2mM dNTPs (NEB), 1x Phusion buffer, 4% DMSO and 0.5U of Phusion polymerase. The following thermal cycling protocol was employed: Denaturation at 98°C for 30s; 20 cycles of denaturation at 98°C (10s), primer annealing at 55°C (30s), extension at 65°C (30s/kb) followed by a final extension cycle at 65°C (300s). Subsequently, the PCR reaction was digested with 2U of DpnI (NEB) to remove methylated parental plasmid DNA. A 2μl aliquot of the reaction was then transformed into the bacterial strain NEB10β (NEB). Lysed bacterial colonies were Sanger-sequenced to confirm the presence of the desired mutation. Plasmid DNA was purified using Qiagen kits according to the manufacturer’s protocol.

### Residue numbering

All amino acid residues of the RTX heavy chain referred to herein are numbered according to the Eu nomenclature [34]. The full length sequences of the Rituximab heavy chains (Isotypes IgG1 a1; IgG1 a2; IgG2; IgG4P & IgG4DM) and light chain can be found in the supplement, S1 Fig and S2 Fig respectively.

### Protein production

Antibodies were expressed using the transient episomal CHO-3E7 expression system [31]. Briefly, plasmid DNA (1μg/ml total transfection volume) was complexed with polyethyleneimmine (PEIPro, Polyplus). DNA-PEI complexes were added to CHO-3E7 in serum-free CHO Freestyle medium (Invitrogen) supplemented with 2mM L-Gln. Typical reaction volumes ranged from 100 – 250ml and transfections were incubated for 7–8 days. The supernatant was harvested by centrifugation and filtration and antibody titers were measured using a Fortebio OctetQK^e^ system in conjunction with protein A biosensors (Pall).

### Protein purification

Antibodies were purified using protein A spin columns (GE Healthcare) according to the manufacturer’s instruction. Protein purity was verified by SDS PAGE and Caliper Labchip GXII protein assay (PerkinElmer). Concentrations were determined by measuring absorbance at 280nm with a Nanodrop photometer (ThermoFisher) utilizing the calculated antibody specific extinction coefficient [35]. High performance size exclusion chromatography (HP-SEC) was used to measure the monomer content of antibody preparations. Briefly, HP-SEC was performed using a Waters 2690 alliance HPLC instrument equipped with a Waters 2487 Dual Absorbance detector employing TSKgel300SWXL gel filtration columns (Tosoh Biosciences). Samples (100–125μg) were run in SEC buffer (200mM L-Arginine, 100mM Na_2_HPO_4_*2H_2_O, pH 6.8), with a flow rate of 1 ml/min. A molecular weight standard (Bio-Rad) and an internal antibody (IgG1) standard were run in parallel to assign peaks. Data analysis and peak quantitation were performed using the internal software package BIChrom Empower (version FR5). Exemplary data can be found in S3 Fig.

### Cell culture

CD20 expressing Ramos cells (ATCC CRL 1596) were cultured in serum-free Ultraculture medium (Lonza) supplemented with 2mM L-Gln. Ramos cells were seeded at 5E5/ml 24h prior to performing apoptosis or ADCC assays. NKL cells [36] were maintained in serum-free Ultraculture medium and supplemented with 2mM L-Gln and 600U/ml Proleukin (Novartis) and were seeded at 1E6/ml 24h prior to performing ADCC assays.

### F(ab)_2_ and Fc preparation

Antibody fragments were prepared using the FragIT cleavage and purification kit (Genovis) according to the manufacturer’s instruction. The kit utilizes the cysteine protease IdeS that cleaves antibodies in the lower hinge region [37].

### Apoptosis assay

**Fig 1:**
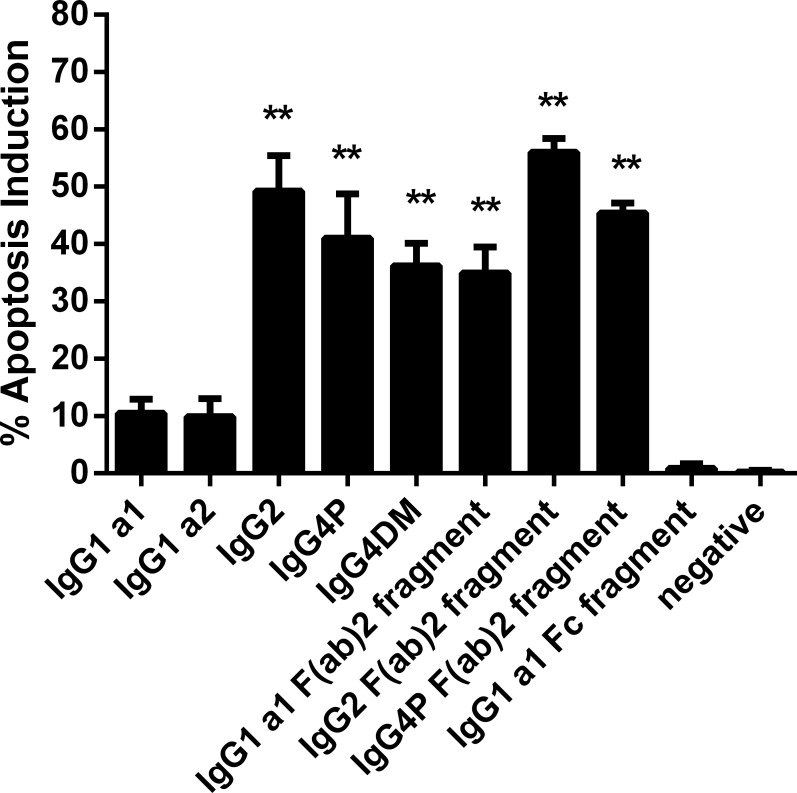
Apoptosis induction potential of different Rituximab isotypes and antibody fragments. The absolute apoptotic induction of different Rituximab isotypes and antibody fragments is shown; full length sequences, numerical values and p-values can be found in the supplemental information. Bars represent the mean of at least three independent assays ± standard deviation. The negative control represents an antibody whose target is not expressed on Ramos cells. P- values were calculated by one-way ANOVA using IgG1 a1 as the control group. ** p≤p0.01.

**Fig 2:**
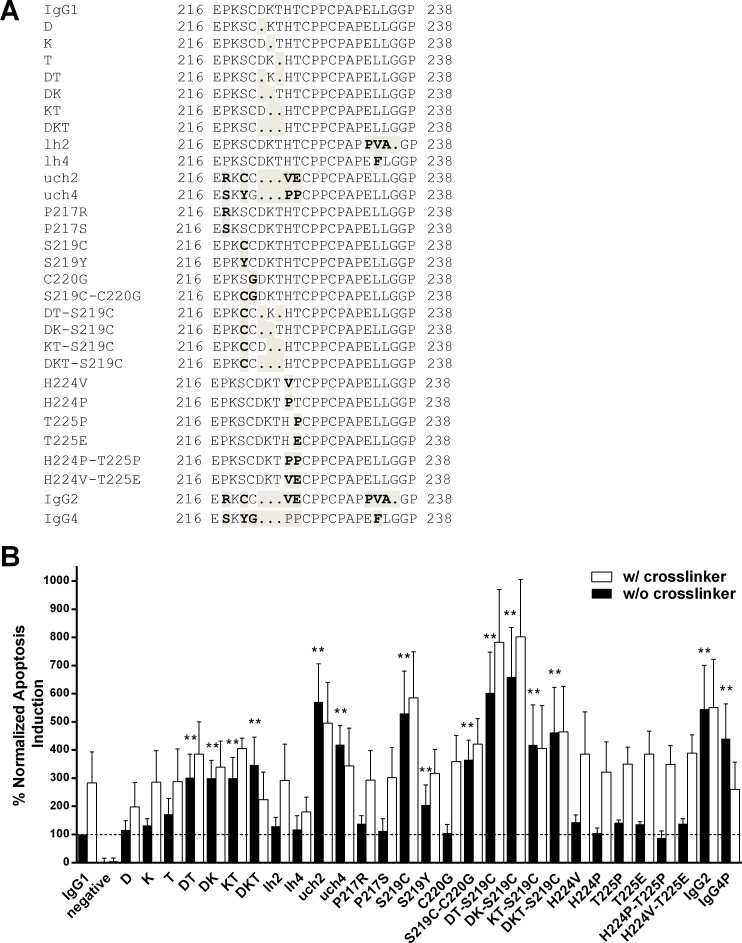
Sequence alignments for the hinge region and normalized apoptosis induction potential of Rituximab hinge variants. (A) The hinge region of the Rituximab heavy chain, from residue 216 to 238 (EU numbering) is shown. Sequences differences between the control IgG1, IgG2, IgG4P and all engineered hinge mutants are bolded and shaded. (B) The bars illustrate the normalized apoptosis values observed for all constructed Rituximab hinge variants in relation to the IgG1 control (100%, dashed line) in the absence (black bars) and presence (white bars) of crosslinking antibody. Values for full length IgG2 and IgG4P are shown as a reference. Reported are the means of at least three independent assays ± standard deviation. P- values were calculated by one-way ANOVA using IgG1 a1 as the control group. ** p ≤ 0.01.

**Fig 3:**
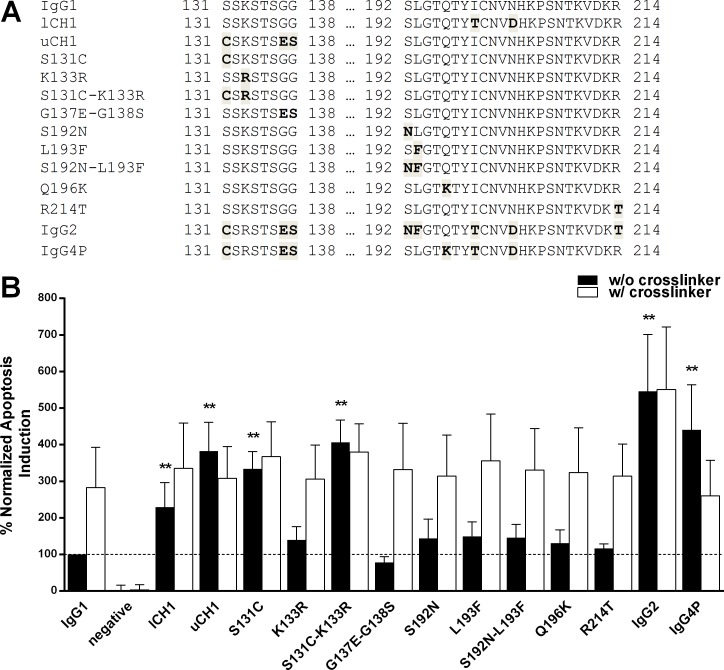
Sequence alignments for the CH1 region of normalized apoptosis induction potential of Rituximab CH1 variants. (A) Two parts of the CH1 region, from residues 131 to 138 and 192 to 214, are illustrated. Sequences differences between the control IgG1, IgG2, IgG4P and all engineered CH1 mutants are shaded. (B) The bars illustrate the normalized apoptosis values observed for all Rituximab CH1 variants in relation to the IgG1 control (100%, dashed line) in the absence (black bars) and presence (white bars) of crosslinking antibody. Values for full length IgG2 and IgG4P are shown as a reference. Shown are the means of at least three independent assays ± standard deviation. ** p ≤ 0.01. The p values that are shown were calculated by one way ANOVA using IgG1 in absence of the crosslinker as the control group.

**Fig 4:**
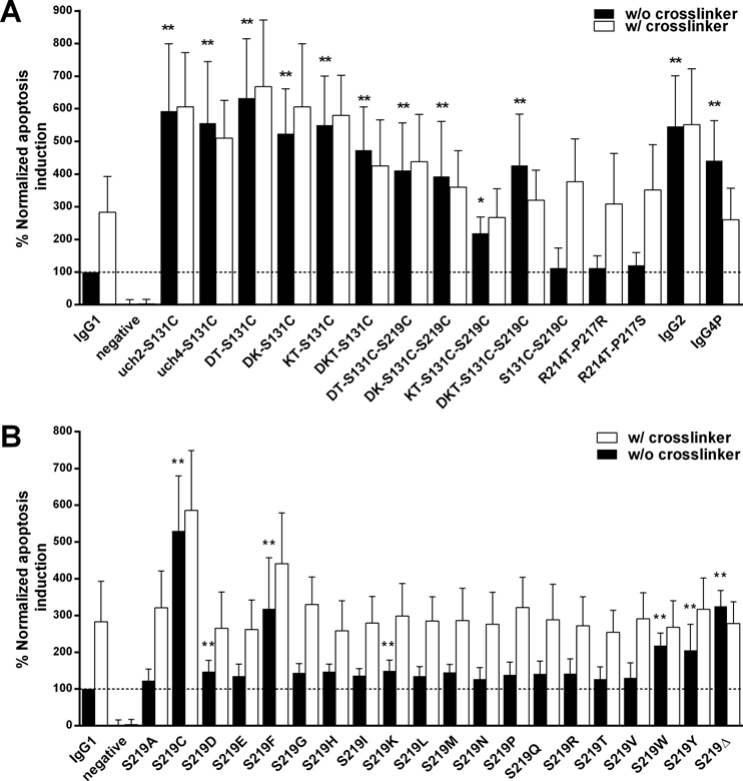
Normalized apoptosis induction potential of Rituximab variants combining hinge and CH1 mutations and mutants covering all possible amino acid exchanges of hinge position 219. (A) The bars illustrate the normalized apoptosis values observed for Rituximab variants that combine hinge and CH1 mutations. Values for full length IgG2 and IgG4P are shown as a reference. (B) shows the impact of all possible amino acid exchanges at hinge position S219. All values are shown in relation to the IgG1 control (100%, dashed line) in the absence (black bars) and presence (white bars) of crosslinking antibody. Data represents the means of at least three independent assays ± standard deviation. * p ≤ 0.05; ** p ≤ 0.01. The shown p values were calculated by one way ANOVA using IgG1 in absence of the crosslinker as the control group.

2000ng of each full length RTX variant, with or without an additional 2000ng (ratio 1:1) of cross-linking antibody (goat-anti-human Fcγ specific, Jackson Immunoresearch) were incubated with 100.000 Ramos cells for 24h in 200μl of Ultraculture medium (Lonza) supplemented with 2mM L-Gln in round-bottom 96w plates at 37°C and 5% CO2. The amount of antibody to use in the apoptosis assay has been empirically determined (see S4 Fig for dose response assessments) to yield a robust readout after 24h. F(ab)_2_ and Fc fragments were used in equimolar amounts equating to 1360ng and 320ng respectively. Following incubation, cells were spun down and 100μl of medium was replaced with 2x AnnexinV binding buffer (BD Pharmigen) containing AnnexinV-FITC and Propidium Iodide (PI; both ebiosciences). Measurement occurred in a FACS Calibur flow cytometer (BD Biosciences) by acquiring 15.000 events per sample and data analysis was performed using the Flowjo software package (Treestar). First, a gate was applied to the forward/side scatter (FSC/SSC) plot. Gated cells were analyzed for AnnexinV-FITC and PI staining patterns. Unstained controls were used to set the gates. Cells negative for both dyes have not undergone apoptosis. Cells showing a PI staining only are considered to be necrotic. In this work the total percentage of apoptosis induction is defined as the sum of AnnexinV-FITC single positive (early apoptotic) and AnnexinV-FITC/PI double positive (late apoptotic) cells. An illustration of the gating strategy can be found in S5 Fig. Spontaneous apoptosis induction in the absence of antibody was subtracted. Reported are absolute (Fig 1) and normalized (Figs 2, 3 & 4) apoptosis induction. For normalization, the value obtained for the IgG1 control of Rituximab in the absence of crosslinking antibody was set to 100% in every experiment. Each antibody variant was measured in at least three independent assays. Further, an IgG1 antibody (Sibrotuzumab) whose target is not present on Ramos cells was used as a negative control [38,39]. Numeric values (mean, standard deviation, N, p-values) are reported in S1 and S2 Tables.

### Cellular Binding Assay

In order to determine a saturation binding curve for RTX IgG1 (S6 Fig), 1E6 Ramos cells per sample were suspended in FACS buffer (PBS, 1% BSA, 0.01% NaN_3_) and incubated for 30 mins at 4°C with serial dilutions of RTX IgG1. Following two washes in FACS buffer, bound antibody was detected using 1μg of a polyclonal FITC (fluorescein isothiocyanate) labelled goat anti human IgG (Jackson Immunoresearch). Samples were measured in a FACS Calibur flow cytometer (BD Biosciences). Data was analyzed by calculating the MFI (median fluorescence intensity) of the vital population using the Flowjo software (Treestar). The EC50 value was obtained by plotting the MFI versus the concentration and applying a 4 parametric logistic dose response model.

**Fig 5:**
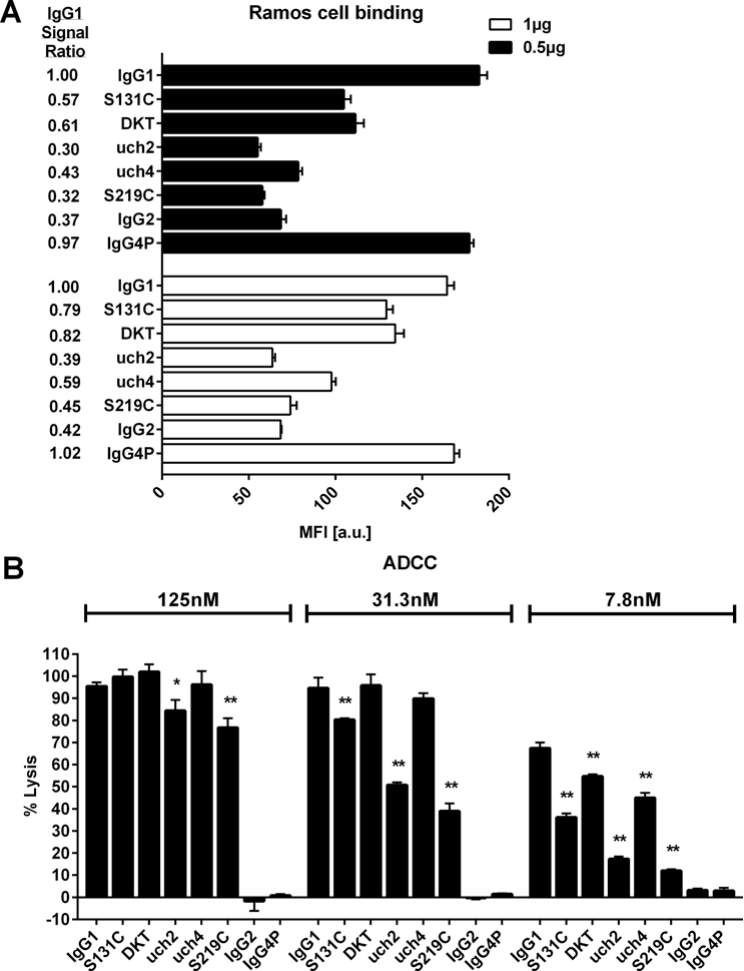
Binding of RTX variants to the surface of Ramos cells and mediation of ADCC via RTX and NKL cells *in vitro*. In (A) the binding of RTX variants to the surface of Ramos cells measured by flow cytometry is shown. Two RTX amounts (1μg, white bars) and 0.5μg (black bars) per 1E6 Ramos cells were used. The IgG1 signal ratio represents the MFI of the RTX variant divided by the MFI of the IgG1 control. (B) shows the result of a RTX specific ADCC assay in which three different concentrations (125nM, 31.3nM, 7.8nM) of the antibodies were incubated with Ramos target and NKL effector cells to evaluate ADCC activity of the RTX variants. * p ≤ 0.05; ** p ≤ 0.01. The p values were calculated by one way ANOVA using IgG1 as a control in each concentration group. Shown are the means of three independent samples ± standard deviation. MFI: median fluorescence intensity; a.u.: arbitrary units.

For direct RTX antibody variant binding (Fig 5A), 1E6 Ramos cells were incubated with 0.5μg (16.7nM) or 1μg (33.3nM) of the respective RTX variant. The amounts were chosen based on the EC50 of RTX binding to Ramos cells (S6 Fig). Bound antibody was detected using 1μg FITC conjugated murine monoclonal anti human kappa light chain antibody (ebioscience).

For competition binding assays (S7 Fig), 1E6 Ramos cells per sample were incubated with 1μg FITC conjugated anti-CD20 antibody clone LT-20 (Santa Cruz Biotechnology) and serial dilutions of the RTX variants. The measured MFI was normalized to the signal obtained in the absence of competing antibody (= 100%) or isotype control stained cells (= 0%). Data points were plotted and fitted using a 4 parametric logistic dose response model to obtain IC50 values.

### ADCC Assay

ADCC assays were performed by employing the CARE-LASS (Calcein-AM Release) assay principle [40]. Briefly, target cells (Ramos) were labelled with 25μM Calcein-AM (Sigma) for 30mins at 37°C in the dark. After washing the labelled cells twice with assay medium (Ultraculture + 2mM L-Gln + 600U/ml Proleukin), Ramos cells were seeded at 10000 cells per well of a 96 well plate and mixed with pre-diluted antibody. NKL cells [36], modified at Boehringer Ingelheim to overexpress CD16-V158, were utilized as effector cells. An effector to target ratio of 30:1 was employed, hence 300000 NKL cells were added to each well. The cells were incubated at 37°C for 3h. Negative (targets and effectors in the absence of antibody to obtain background lysis values) and positive controls (targets and effectors in the presence of 1% Triton X100 (Sigma) to obtain maximum lysis values) were included in each assay. Following incubation, the plates were centrifuged and 100μl of supernatant were transferred to a new black microtiter plate and measured in an Infinite M200Pro (Tecan) microplate reader using standard fluorescein settings (excitation 480nm, emission 520nm, gain 100, 10 flashes). The specific lysis was calculated as follows: 100% * (observed lysis–background lysis)/(maximum lysis–background lysis).

### Figures and Statistics

All figures were created with Prism v6 (Graphpad, USA), which was also used for statistical evaluation of the data set. All p-values in this work were computed by one-way ANOVA followed by the Dunnett’s test. The respective RTX IgG1 reference served as a control group.

## Results

### IgG2 or IgG4 versions of Rituximab are more effective at inducing apoptosis *in vitro* than IgG1

Rituximab comprises murine variable heavy and light chain sequences in combination with human constant domains of the kappa light chain and IgG1 heavy chain isotypes. We have observed that reformatting the RTX heavy chain into IgG2 or IgG4 isotypes drastically impacts the antibody’s ability to induce direct apoptotic cell death in a CD20 specific *in vitro* assay using the human Burkitt’s lymphoma cell line Ramos (Fig 1; S1 Table). Whereas the two IgG1 allotypic variants IgG1 a1 and IgG1 a2 (a1 (G1m1): D356, L358; a2 (nG1m1): E356; M358) induced apoptosis in only 11% or 10% of all cells after 24h in culture respectively, an IgG2 version of RTX boosted this value to 49%. Two stabilized IgG4 variants of the same antibody, IgG4P and IgG4DM (carrying S228P and S228P-L235E exchanges) [41,42] caused apoptosis in 41% and 36% of the Ramos cell population.

Next, we cleaved IgG1 a1, IgG2 and IgG4P into F(ab)_2_ and Fc fragments using the IdeS protease [37]. As observed for the full length IgGs, the F(ab)_2_ fragment derived from IgG2 induced the highest level of apoptosis (56%), followed by the IgG4P F(ab)_2_ (45%). The IgG1 a1 F(ab)_2_ fragment caused apoptosis in 35% of all Ramos cells, a level three times higher than seen for the full length IgG1 a1 antibody. The Fc fraction from IgG1 a1 was tested as a control. As expected, the Fc fragment, not containing the antigen binding region, did not induce apoptosis.

### Modifying Rituximab’s IgG1 hinge region to mimic IgG2 or IgG4 impacts apoptosis induction

Some of the most striking structural differences between IgG1, IgG2 and IgG4 are located in the hinge region (Fig 2A). The hinge itself is split in three regions, the upper; core and lower hinge [43]. The upper hinge, residues 216 to 225 according to the Eu nomenclature [34], comprises the sequence motif EPKSCDKTHT in IgG1. Notably, both IgG2 and IgG4 upper hinges are shorter than seen for IgG1. IgG2’s upper hinge contains 3 amino acid residues (ERK), whereas the upper hinge region of IgG4 is built by 7 amino acids (ESKYGPP). In case of the core hinge (residues 226–230), IgG1 and IgG4 both comprise 5 amino acids; CPPCP for IgG1 and CPSCP for wild type IgG4. A common IgG4 engineering strategy to avoid the formation of half molecules [41] is substituting the serine residue at position 228 with a proline and has also been employed for the IgG4P variant used herein. In contrast, the IgG2 core hinge is longer and contains an additional 4 amino acids (CCVECPPCP). Finally the lower hinge region (residues 231–238) is similar between IgG1 (APELLGGP) and IgG4 (APEFLGGP) apart from a L234F exchange. IgG2 exhibits additional changes in the region (APPVAGP), with two amino acid replacements (L234V and L235A) and a deletion of the glycine at position 236.

In order to evaluate which residues where responsible for IgG2 and IgG4’s enhanced apoptosis potential; we constructed a selection of hybrid IgG1 based antibodies (Fig 2A) containing substitutions in the hinge region mimicking the respective IgG2 and IgG4 residues and evaluated them for apoptosis induction (Fig 2B).

One notable hallmark of the IgG2 and IgG4 upper hinges is the lack of the motif DKT. Therefore we deleted one, two or all three amino acids in the RTX IgG1 heavy chain. The exclusive deletion of D, K or T has only a small, not statistically significant, impact on the apoptosis potential. Deleting two residues simultaneously, however, results in a 3 fold increase of observed cell death (302% for DT; 300% for DK and 300% for KT). Lastly, when removing the entire motif (mutant DKT) apoptosis levels of 347% were achieved.

Transplanting the entire lower hinges of IgG2 and IgG4 onto the RTX IgG1 heavy chain (variants lh2 and lh4) had no effect on observed apoptosis levels, suggesting the lower hinge is not involved in eliciting direct cell death.

Next, we assessed the transplantation of the complete upper and core hinges of IgG2 and IgG4 onto the IgG1 heavy chain backbone. The mutants uch2 (571%) and uch4 (419%) showed greatly enhanced apoptosis potential that was comparable to the values observed for antibodies possessing full length IgG2 (546%) and IgG4P (441%) heavy chains.

As uch2 and uch4 contain 7 or 8 amino acid changes respectively (Fig 2A), we wanted to assess what impact individual mutations–beyond the DKT motif described before–would have on RTX’s apoptotic potential. A variety of the single or double exchanges had no or only a moderate effect that proved statistically insignificant. P217R, P217S, C220G, H224V, H224P, T225P, T225E, H224P-T225P and H224V-T225E did not have an impact. One residue, however, the serine at position 219 of an IgG1 that is changed to a cysteine in IgG2 and a tyrosine in IgG4, had a large influence. The RTX mutant S219C showed an apoptosis induction potential of 530%, whereas S219Y boosted observed cell death levels to 205%. A S219C-C220G combination yielded 366%. Therefore the amino acid at position 219 appears to be a key modulator of RTX mediated direct cell death.

Further, we combined the S219C substitution with the double (DT, DK, KT) or triple (DKT) deletions. Variants DT-S219C and DK-S219C showed the strongest apoptosis induction potential with 603% and 660% respectively. KT-S219C achieved 418% and DKT-S219C resulted in 463% apoptosis induction. Adding the S219C exchange enhanced apoptosis potential of all double and triple deletions (p<0.05). The combinations, however, did not differ significantly from using the S219C substitution alone, reinforcing the importance of the residue at position 219 for effective apoptosis induction.

It has been described that crosslinking antibodies directed against the Fc domain can enhance apoptosis induction by RTX *in vitro* [8]. We have tested all antibodies in the absence and presence of such a crosslinking secondary antibody. The antibody to crosslinker ratio employed was 1:1. For the IgG1 control, the crosslinker boosted the observed apoptosis levels around three-fold (Fig 2B, white bars). Notably, no such crosslinking effect could be observed for full length IgG2 and IgG4P. In case of IgG4P, the induced levels of apoptosis were reduced when the crosslinker was present. Interestingly, the Rituximab IgG1 mutants encompassing key structural features of the IgG2 or IgG4 hinges, that, on their own, boost apoptosis, cannot be further enhanced by crosslinking (e.g. variants DKT, uch2, uch4, S219C, KT-S219C, DKT-S219C). All other variants retained the apoptosis enhancing crosslinking effect, though the level of cell death increase observed was lower in some cases (e.g. DT, DK, KT, lh4, S219Y, DT-S219C, DK-S219C).

### Modulating Rituximab apoptosis potential by engineering IgG2 and IgG4 specific CH1 residues

IgG1, IgG2 and IgG4 heavy chain sequences are further set apart by differences in their CH1 domain (Fig 3A). As direct apoptosis induction by anti CD20 antibodies is considered to be an effect primarily mediated by the F(ab)_2_ fragment [7], we hypothesized that sequence variations in the CH1 domain may also be of key importance in order to explain the observed enhanced apoptosis potential of IgG2 and IgG4.

IgG2 and IgG4 are characterized by identical sequences in an upper part of the CH1 region, between residues 131 and 138. Compared to IgG1, 4 amino acids differ. The serine at position 131, the lysine at 133 and two glycines at 137 and 138 are replaced with cysteine, arginine, glutamic acid and serine respectively. In the lower part of the CH1 domain (residues 192–214), both IgG2 and IgG4 have a threonine in position 199 instead of IgG1’s isoleucine and an aspartic acid in position 203 replacing asparagine. Further hallmarks of an IgG2 CH1 are asparagine and phenylalanine in positions 192 and 193 (as opposed to IgG1’s serine and leucine) and threonine in position 214 (instead of arginine). The IgG4 CH1 region is also identified by a lysine in position 196 replacing the IgG1 glutamine.

As before, we expressed hybrid IgG1 based RTX antibodies containing several of the IgG2 and/or IgG4 CH1 hallmark residues and assessed them *in vitro* concerning their apoptosis induction potential (Fig 3B). A combination of two lower CH1 exchanges (variant lCH1, I199T and N203D) boosted apoptosis induction to 205% compared to the IgG1 control. The variants S192N, L193F, Q196K, the combination S192N-L193F and R214T had no significant impact.

The antibody termed uCH1, containing upper CH1 exchanges S131C, G137E and G137S, achieved 383%. S131C on its own boosts apoptosis levels to 334% and in conjunction with K133R (S131C-K133R) to 406% compared to the IgG1 control while K133R alone had no significant influence. Thus, the residue at position 131 was identified as the key driver of RTX apoptosis ability.

Crosslinking did not further enhance observed cell death for uCH1 or S131C containing mutants, but did for all other tested variants.

### Combining amino acid exchanges in the hinge and CH1 domains can maximize RTX’s apoptosis induction potential

Finally, having identified key residues involved in RTX mediated apoptosis induction in both the hinge and CH1 region, we wanted to assess the impact of combinatorial mutations (Fig 4A).

Combining the hinge variants uch2 and uch4 with S131C resulted in observed mean apoptosis levels of 593% (uch2-S131C) and 556% (uch4-S131C). Both combinations’ effectiveness is comparable to either uch2 and uch4 alone as well as full length IgG2 and IgG4P (p>0.05), indicating that the presence of the upper and core hinges is sufficient for obtaining IgG2 and IgG4P like apoptotic behavior and that there is no further benefit of adding S131C. Neither uch2-S131C nor uch4-S131C could be enhanced by crosslinking.

Combining the double or triple deletions of the hinge DKT motif with the CH1 S131C exchange had a beneficial impact on apoptosis induction. The combinatorial mutants DT-S131C (633%), DK-S131C (524%), KT-S131C (550%) and DKT-S131C (473%) were all able to elicit higher apoptosis levels than the amino acid changes did in isolation (all p-values <0.05).

Adding the critical hinge variation S219C to the mix however, did not further increase the observed levels of cell death. DT-S131C-S219C (411%), DK-S131C-S219C (393%), KT-S131C-S219C (219%) and DKT-S131C-S219C (427%) had a significant improved apoptosis induction potential in comparison the IgG1 wild-type, but did not reach the same levels as seen without the addition of S219C. None of these variants benefited from cross-linking.

In addition, the combination of S131C-S219C, the two hinge and CH1 variations which each strongly enhance apoptotic potential when used exclusively do not have a beneficial impact when used together.

Two further variants, R214T-P217R and R214T-P217S, were investigated. However, neither combination impacted apoptosis induction in relation to the IgG1 control, but were boosted in presence of the crosslinker.

### A cysteine at hinge position 219 increases RTX apoptosis induction potential more than any other possible single exchange

After having previously identified the S219C hinge exchange, a hallmark of IgG2 heavy chain structure, as a key driver in RTX apoptosis potential, we created RTX variants carrying all possible amino acids in the same position (Fig 4B). While none of the variants exhibit the same apoptotic strength as S219C, S219D (147%) and S219K (149%) result in small increases that are statistically significant. Placing the aromatic amino acids phenylalanine (S219F: 318%) or tryptophan (S219W: 218%) in position 219 enhances RTX apoptotic ability–which is in the line with the results obtained for S219Y (205%), the structural feature of IgG4 hinges. Deleting the serine at position S219 (variant S219Δ: 325%) also enhanced observed apoptosis levels. All other exchanges had no noticeable impact.

### Cellular binding levels of RTX on Ramos cells correlate to the antibody isotype

When assessing the binding levels of a selection of RTX variants on the surface of Ramos cells by flow cytometry using non-saturating antibody amounts (Fig 5A, S6 Fig), it became apparent that the antibody isotype correlates to the signal strength. We employed a kappa light chain specific secondary antibody in order to avoid any interference of detecting the CD20 bound antibodies through the structurally variable IgG heavy chain. When looking at the IgG1 signal ratio (MFI of RTX variant divided by MFI of IgG1 control), all antibodies with the exception of IgG4P resulted in lower signals equating a lower number of antibody molecules bound to the cell. Notably, this happens despite the RTX variant’s comparable affinities evidenced by their similar ability to compete with a directly labelled CD20 antibody (S7 Fig). The effect is most drastic for full length IgG2 (ratio = 0.37 for 0.5μg and 0.42 for 1μg) and the variant uch2 carrying the entire upper and core hinges derived from IgG2 (ratio = 0.32 for 0.5μg and 0.39 for 1μg). Similarly, the single point mutation S219C, encompassing a major hallmark of the IgG2 hinge resulted in ratios of 0.32 (0.5μg) and 0.45 (1μg). The CH1 mutant S131C (ratios 0.57 and 0.79) and the DKT hinge deletion (ratios 0.61 and 0.82) also caused a, albeit less drastic, reduction of the number of RTX molecules bound to the cell surface. Curiously, IgG4P showed no such reduction, but the mutant uch4, which encompasses the IgG4P derived upper and core hinge sequence did (ratios = 0.43 for 0.5μg and 0.59 for 1μg).

### RTX variants with enhanced apoptosis potential exhibited reduced ADCC induction

Lastly, we assessed the ADCC potential of a number of RTX variants with enhanced apoptosis potential (Fig 5B). A CARE-LASS assay [40] measuring the amount of released fluorescing calcein from lysed CD20 expressing Ramos cells in the presence of antibody and the effector cell line NKL [36] was employed. As expected, neither IgG2 nor IgG4P induced ADCC at any of the utilized concentrations (125nM, 31.3nM and 7.8nM). All of the RTX variants assessed, S131C, DKT, uch2, uch4 and S219C were able to elicit ADCC, however their ability to do so is reduced in comparison to the IgG1 control. The strongest impact is seen for variants uch2 and S219C, with reduced ADCC induction already apparent at the highest concentration of 125nM. uch4 and DKT show a smaller reduction in ADCC potential, with effects becoming apparent at 31.3nM and 7.8nM respectively. Overall, the hybrid IgG1 antibodies that encompass structural features of IgG2 and IgG4P were less able to elicit ADCC than a full length IgG1, but still had some ADCC potential left unlike the parental antibodies IgG2 and IgG4P that were not able to mediate cell lysis via ADCC at all.

## Discussion

The induction of apoptosis in target cells by therapeutic antibodies is a key mode of action for eliminating tumor cells in cancer [6]. Rituximab has been shown to elicit programmed cell death both *in vitro* [8,9,15] and *in vivo* [14] and is established as a valuable treatment option in B cell driven malignancies [44,45]. However, many patients treated with Rituximab become refractory or relapse eventually which has triggered a large body of work focusing on the generation of CD20 antibodies with improved efficacy. To date, this work has largely aimed at enhancing ADCC or CDC and the creation of antibodies against different CD20 epitopes [5,46,47]. Our results, showing that RTX IgG2 and IgG4 isotypes are more effective at eliciting direct apoptotic cell death than IgG1, open up an additional engineering route. To our knowledge, this is the first study systematically investigating the apoptotic ability of human IgG2 and IgG4 isotypes in relation to the more commonly used IgG1.

We found that RTX formatted as IgG2 or IgG4 induced about 5- and 4-fold more apoptosis in CD20 expressing Ramos cells than RTX IgG1. The exact mechanisms behind this behavior are currently being investigated, but recent advances in the understanding of CD20 antibodies may offer relevant clues. CD20 antibodies have been classified as either type I or type II based on their functional abilities [48]. Type I antibodies, such as Rituximab, are able to translocate CD20 into detergent-insoluble lipid rafts and cause strong CDC but weaker apoptosis. Type II CD20 antibodies display an inverse behavior with a reduced ability to form lipid rafts, weak CDC and strong apoptosis [9,11,49]. Further, CD20 binding modes and geometries appear to be different, with B cells only binding half as many type II molecules as type I [11,49,50].

So far, the type I/II behavior has been largely attributed to the recognition of distinct but overlapping CD20 epitopes and not the antibody isotype [51,52]. It has, however, been reported that changing an antibody’s IgG isotype while keeping the variable regions constant can impact the antigen binding characteristics [53]. Our data suggests that by changing the isotype to IgG2 or IgG4, Rituximab’s potential to induce direct apoptotic cell death can be pushed from type I towards type II behavior.

Accordingly, the same effect can be obtained by transplanting hallmark IgG2/4 residues from the upper and core hinge region or CH1 domain, and presumably the contained structural features, onto an otherwise IgG1 like heavy chain. Hence, the hinge and CH1 domains are clearly of key importance in mediating RTX’s apoptotic ability.

As with the stoichiometric binding data obtained from model type I/II antibodies [11,49,50], we also observed that fewer RTX IgG2 molecules and hybrid IgG1 RTX constructs carrying IgG2/4 structural features bind to the CD20 bearing Ramos surface than seen for the standard RTX IgG1. This supports the theory that the hybrid antibodies shift towards type II binding and presumably functional behavior. RTX IgG4P binding however, proved the exception as there were no differences in comparison to IgG1. It appears additional mechanisms are involved that require further investigation.

One further hypothesis helping to explain our data may be the current knowledge of overall IgG structure and flexibility, in which the hinge region plays an important role. The upper hinge is particularly relevant, with shorter upper hinges resulting in increased rigidity of the antibody [54]. Consequently, the IgG1 hinge is the most flexible and the IgG2 hinge the most rigid. IgG4 exhibits intermediate flexibility. In addition, IgG2 (127°) and IgG4 (128°) exhibit wider F(ab)-F(ab) angles than IgG1 (117°) [54]. These differences and the resulting conformational states are held responsible for the IgG isotypes’ differential abilities to bind and activate complement proteins and Fcγ receptors [55–57].

In light of this work, it appears that Rituximab versions with more rigid hinges and wider F(ab)-F(ab) angles are more potent at inducing direct cell death. Also, the F(ab)_2_ fragment derived from RTX IgG1, induces higher levels of apoptosis than the full length antibody, possibly due to being able to adopt a wider F(ab)-F(ab) angle when not constrained by the Fc domain. Results from the structural investigation of CD20 antibodies and their respective binding modes support this hypothesis. Crystallography studies and epitope mapping have revealed that type I and type II antibodies bind CD20 in different orientations and essentially stabilize different CD20 subpopulations in the cell membrane, which, in turn, results in differential intracellular signaling processes [51,58]. A proposed binding model suggests that each type I antibody binds between two CD20 membrane tetramers, whereas each type II antibody binds within one tetramer [51]. In the type II CD20 antibody GA101, which binds a RTX overlapping epitope, type II behavior could be linked to a leucine to valine exchange in the elbow-hinge region causing an angle that is 30° wider than seen for classic type I antibodies [49]. Reverting to leucine and presumably a narrower elbow hinge angle caused GA101 to adopt a type I phenotype of reduced apoptotic potential [49,58].

In general, our hybrid antibodies exhibited stronger apoptosis modulation when more than one amino acid was exchanged for the corresponding IgG2 or IgG4 sequence (e.g. transplantation of the entire upper and core hinges had a higher impact than replacing individual residues). Notable exceptions are positions 131 and 219, where the replacement of the IgG1 serine with the cysteine found in IgG2 or IgG4 on its own has a drastic benefit. In IgG2 and IgG4 these cysteines are involved in disulfide bond formation [59,60]. It is therefore conceivable that the IgG1 variants carrying these cysteines are able for form additional stabilizing inter- or intra-chain disulfide bonds enabling the antibody to adopt a conformation conducive to apoptosis induction upon CD20 binding. However, the combination of S131C and S219C exchanges in a RTX variant failed to enhance apoptosis further. The S131C-S219C mutant did not differ from the IgG1 control and, when combining S131C-S219C with deletion variants of the DKT hinge motif, the presence of both cysteines was not beneficial. Thus it is possible that when there are two additional cysteines in the CH1 and hinge region (compared to IgG1), the antibody adopts a structure that is less favorable as far as apoptosis induction is concerned.

The disulfide bond structure of human IgG2 has been a particular focus of scrutiny in the literature. IgG2 is known to form multiple disulfide bond isoforms: IgG2A, IgG2B and IgG2A/B whose presence and equilibrium can be influenced by diverse parameters such as cell culture conditions, thermal stress or the presence of reducing agents [61]. Interestingly, White et al [62] have recently been able to show that hybrid IgG1 molecules carrying the IgG2 hinge and CH1 domains directed against co-stimulatory molecules such as CD40 possessed superagonistic properties independent of Fcγ receptor engagement in comparison to normal IgG1. The authors were able to attribute this behavior to the alternative IgG2B disulfide bond isomer fraction and hypothesize that the compact IgG2B conformation allows close crosslinking of the target receptor and subsequent enhanced signaling. Future experiments will reveal whether our hybrid antibodies are also able to adopt an IgG2B like disulfide bonding status and cause increased clustering of CD20 followed by efficient signaling that is responsible for observed apoptosis levels.

Taken together, the existing knowledge about the binding mode of anti-CD20 antibodies and IgG2/4 structure in general allows us to propose a binding model for our apoptosis enhanced RTX variants, which is similar to the hypothesis described by Cragg [63]. RTX IgG1 and hybrid variants showing unaltered apoptosis induction potential, as classic type I CD20 antibodies, exhibit CD20 inter-tetramer binding that remains amenable to additional crosslinking by the secondary antibody. Full length RTX IgG2/IgG4P and hybrid IgG1 variants carrying the key CH1/hinge residues from those scaffolds–due to adopting wider Fab-Fab angles—acquire the ability for CD20 intra-tetramer binding which results in reduced overall binding capacity but enhanced apoptosis induction. According to our data, this proposed binding mode is no longer amenable to crosslinking via the secondary antibody, possibly due to conformational changes and steric hindrance. It is further conceivable that the polyclonal secondary goat anti-human Fcγ antibody has reduced ability to bind to antibodies containing IgG2/IgG4 like hinges or, if it does bind, prevents crosslinked complexes from optimal interaction with the target. The validation of this proposed binding model using structural biology will be the focus of a follow-up study.

Our data also shows that the increase in apoptosis potential comes at the price of lower *in vitro* ADCC activity. The hybrid IgG1 antibodies encompassing key structural features from IgG2/4 exhibited a reduced ability to induce ADCC in Ramos cells using the NK derived effector cell line NKL. As IgG2 and IgG4 are known to not be able to induce NK mediated ADCC, this finding is not surprising and further implies that the hybrid antibodies adopt parts of the IgG2/4 structure. Nevertheless, as the hybrid antibodies maintained some degree of the IgG1’s ADCC ability, it is possible that a combination of the apoptosis increasing mutants with an ADCC enhancement technology, such as removal of the core fucose [64,65] or CH2 engineering [66], may result in novel antibodies with both optimal ADCC and apoptosis function.

Notably, all hybrid antibodies constructed in this work could be expressed in CHO cells with normal yields, purified via standard Protein A chromatography and presented with expected heavy and light chain patterns during SDS PAGE and comparable levels of monomer when analyzed by HP-SEC (S3 Fig). Hence the engineering of the CH1 and hinge domains, particularly the cysteine exchanges that potentially influence the disulfide bond formation process, did not unduly impact overall protein stability. Further investigations into the detailed CMC properties and disulfide structures of the hybrid IgG1 antibodies are presently underway.

In conclusion, we have presented evidence that formatting Rituximab as IgG2 or IgG4 isotypes has a beneficial effect on the direct induction of apoptotic cell death *in vitro*. The responsible structural features for this behavior are located in the CH1 and hinge domains of the heavy chain. Individual IgG2 or IgG4 hallmark residues can be introduced into an otherwise IgG1 antibody which leads to an improved apoptotic performance. Our data indicates that an altered binding geometry to CD20 is at least partially responsible for the observed behavior, possibly due to the hybrid antibodies presenting different conformations. Whether this coincides with changes of CD20 mediated signaling needs further exploration. The transferability of the engineering principle to antibodies directed against other targets requires more investigation, as does the question whether the increased apoptotic cytotoxicity established *in vitro* using the Ramos cell translates to improved efficacy *in vivo*, where antibody mediated target cell killing is a multifaceted process involving additional Fc receptor bearing cell lineages. Further, it would be interesting to assess whether the hybrid antibodies possess an altered immunogenicity potential. While the hybrid hinges and CH1 domains should not create neo-antigens as all motifs are taken from naturally occurring IgG2 and IgG4 antibodies, it remains to be seen whether the introduction of such motifs into an IgG1 causes the antibody to be more immunogenic.

Overall, the presented work adds an interesting protein engineering design option for therapeutic antibodies whose ability to kill target cells is directly linked to their efficacy, such as in oncology.

## Supporting Information

S1 Fig Full length alignment of Rituximab heavy chains in IgG1 a1; IgG1 a2; IgG2; IgG4P and IgG4DM format. (DOCX)

S2 Fig Full length sequence of Rituximab light chain. (DOCX)

S3 Fig Representative purity analysis of antibody preparations by HP-SEC. (PDF)

S4 Fig Dose Response analysis of apoptosis induction. (PDF)

S5 Fig Flow cytometry gating strategy employed to identify apoptotic Ramos cells. (PDF)

S6 Fig RTX IgG1 saturation binding curve. (PDF)

S7 Fig Competition binding assay for RTX variants. (PDF)

S1 Table Absolute apoptotic activity values for different IgG isotypes and fragments. (DOCX)

S2 Table Normalized apoptotic activity values. (DOCX)

## Abbreviations

RTX: Rituximab
CDC: complement dependent cytotoxicity
ADCC: antibody dependent cellular cytotoxicity
HC: heavy chain
LC: light chain
mAb: monoclonal antibody
