# Supplementary material for: Reformatting Rituximab into Human IgG2 and IgG4 Isotypes Dramatically Improves Apoptosis Induction In Vitro
Source: PLoS One. 2015 Dec 29;10(12):e0145633. doi: 10.1371/journal.pone.0145633 (PMC4694715; doi:10.1371/journal.pone.0145633)
Supplement: S1 Fig — (DOCX) [file pone.0145633.s001.docx]

1 50

IgG1_a1 QVQLQQPGAE LVKPGASVKM SCKASGYTFT SYNMHWVKQT PGRGLEWIGA

IgG1_a2 QVQLQQPGAE LVKPGASVKM SCKASGYTFT SYNMHWVKQT PGRGLEWIGA

IgG2 QVQLQQPGAE LVKPGASVKM SCKASGYTFT SYNMHWVKQT PGRGLEWIGA

IgG4P QVQLQQPGAE LVKPGASVKM SCKASGYTFT SYNMHWVKQT PGRGLEWIGA

IgG4DM QVQLQQPGAE LVKPGASVKM SCKASGYTFT SYNMHWVKQT PGRGLEWIGA

51 100

IgG1_a1 IYPGNGDTSY NQKFKGKATL TADKSSSTAY MQLSSLTSED SAVYYCARST

IgG1_a2 IYPGNGDTSY NQKFKGKATL TADKSSSTAY MQLSSLTSED SAVYYCARST

IgG2 IYPGNGDTSY NQKFKGKATL TADKSSSTAY MQLSSLTSED SAVYYCARST

IgG4P IYPGNGDTSY NQKFKGKATL TADKSSSTAY MQLSSLTSED SAVYYCARST

IgG4DM IYPGNGDTSY NQKFKGKATL TADKSSSTAY MQLSSLTSED SAVYYCARST

101 150

IgG1_a1 YYGGDWYFNV WGAGTTVTVS AASTKGPSVF PLAPSSKSTS GGTAALGCLV

IgG1_a2 YYGGDWYFNV WGAGTTVTVS AASTKGPSVF PLAPSSKSTS GGTAALGCLV

IgG2 YYGGDWYFNV WGAGTTVTVS AASTKGPSVF PLAPCSRSTS ESTAALGCLV

IgG4P YYGGDWYFNV WGAGTTVTVS AASTKGPSVF PLAPCSRSTS ESTAALGCLV

IgG4DM YYGGDWYFNV WGAGTTVTVS AASTKGPSVF PLAPCSRSTS ESTAALGCLV

151 200

IgG1_a1 KDYFPEPVTV SWNSGALTSG VHTFPAVLQS SGLYSLSSVV TVPSSSLGTQ

IgG1_a2 KDYFPEPVTV SWNSGALTSG VHTFPAVLQS SGLYSLSSVV TVPSSSLGTQ

IgG2 KDYFPEPVTV SWNSGALTSG VHTFPAVLQS SGLYSLSSVV TVPSSNFGTQ

IgG4P KDYFPEPVTV SWNSGALTSG VHTFPAVLQS SGLYSLSSVV TVPSSSLGTK

IgG4DM KDYFPEPVTV SWNSGALTSG VHTFPAVLQS SGLYSLSSVV TVPSSSLGTK

201 250

IgG1_a1 TYICNVNHKP SNTKVDKRVE PKSCDKTHTC PPCPAPELLG GPSVFLFPPK

IgG1_a2 TYICNVNHKP SNTKVDKRVE PKSCDKTHTC PPCPAPELLG GPSVFLFPPK

IgG2 TYTCNVDHKP SNTKVDKTVE RKCC...VEC PPCPAPPVAG .PSVFLFPPK

IgG4P TYTCNVDHKP SNTKVDKRVE SKYG...PPC PPCPAPEFLG GPSVFLFPPK

IgG4DM TYTCNVDHKP SNTKVDKRVE SKYG...PPC PPCPAPEFEG GPSVFLFPPK

251 300

IgG1_a1 PKDTLMISRT PEVTCVVVDV SHEDPEVKFN WYVDGVEVHN AKTKPREEQY

IgG1_a2 PKDTLMISRT PEVTCVVVDV SHEDPEVKFN WYVDGVEVHN AKTKPREEQY

IgG2 PKDTLMISRT PEVTCVVVDV SHEDPEVQFN WYVDGVEVHN AKTKPREEQF

IgG4P PKDTLMISRT PEVTCVVVDV SQEDPEVQFN WYVDGVEVHN AKTKPREEQF

IgG4DM PKDTLMISRT PEVTCVVVDV SQEDPEVQFN WYVDGVEVHN AKTKPREEQF

301 350

IgG1_a1 NSTYRVVSVL TVLHQDWLNG KEYKCKVSNK ALPAPIEKTI SKAKGQPREP

IgG1_a2 NSTYRVVSVL TVLHQDWLNG KEYKCKVSNK ALPAPIEKTI SKAKGQPREP

IgG2 NSTFRVVSVL TVVHQDWLNG KEYKCKVSNK GLPAPIEKTI SKTKGQPREP

IgG4P NSTYRVVSVL TVLHQDWLNG KEYKCKVSNK GLPSSIEKTI SKAKGQPREP

IgG4DM NSTYRVVSVL TVLHQDWLNG KEYKCKVSNK GLPSSIEKTI SKAKGQPREP

351 400

IgG1_a1 QVYTLPPSRD ELTKNQVSLT CLVKGFYPSD IAVEWESNGQ PENNYKTTPP

IgG1_a2 QVYTLPPSRE EMTKNQVSLT CLVKGFYPSD IAVEWESNGQ PENNYKTTPP

IgG2 QVYTLPPSRE EMTKNQVSLT CLVKGFYPSD IAVEWESNGQ PENNYKTTPP

IgG4P QVYTLPPSQE EMTKNQVSLT CLVKGFYPSD IAVEWESNGQ PENNYKTTPP

IgG4DM QVYTLPPSQE EMTKNQVSLT CLVKGFYPSD IAVEWESNGQ PENNYKTTPP

401 450

IgG1_a1 VLDSDGSFFL YSKLTVDKSR WQQGNVFSCS VMHEALHNHY TQKSLSLSPG

IgG1_a2 VLDSDGSFFL YSKLTVDKSR WQQGNVFSCS VMHEALHNHY TQKSLSLSPG

IgG2 MLDSDGSFFL YSKLTVDKSR WQQGNVFSCS VMHEALHNHY TQKSLSLSPG

IgG4P VLDSDGSFFL YSRLTVDKSR WQEGNVFSCS VMHEALHNHY TQKSLSLSLG

IgG4DM VLDSDGSFFL YSRLTVDKSR WQEGNVFSCS VMHEALHNHY TQKSLSLSLG

451

IgG1_a1 K

IgG1_a2 K

IgG2 K

IgG4P K

IgG4DM K

**S1 Fig Full length alignment of Rituximab heavy chains in IgG1 a1; IgG1 a2; IgG2; IgG4P and IgG4DM format**
