# Supplementary material for: Reformatting Rituximab into Human IgG2 and IgG4 Isotypes Dramatically Improves Apoptosis Induction In Vitro
Source: PLoS One. 2015 Dec 29;10(12):e0145633. doi: 10.1371/journal.pone.0145633 (PMC4694715; doi:10.1371/journal.pone.0145633)
Supplement: S2 Fig — (DOCX) [file pone.0145633.s002.docx]

1 50

QIVLSQSPAI LSASPGEKVT MTCRASSSVS YIHWFQQKPG SSPKPWIYAT

51 100

SNLASGVPVR FSGSGSGTSY SLTISRVEAE DAATYYCQQW TSNPPTFGGG

101 150

TKLEIKRTVA APSVFIFPPS DEQLKSGTAS VVCLLNNFYP REAKVQWKVD

151 200

NALQSGNSQE SVTEQDSKDS TYSLSSTLTL SKADYEKHKV YACEVTHQGL

201

SSPVTKSFNR GEC

**S2 Fig Full length sequence of Rituximab light chain**
