# Supplementary material for: Reformatting Rituximab into Human IgG2 and IgG4 Isotypes Dramatically Improves Apoptosis Induction In Vitro
Source: PLoS One. 2015 Dec 29;10(12):e0145633. doi: 10.1371/journal.pone.0145633 (PMC4694715; doi:10.1371/journal.pone.0145633)
Supplement: S3 Fig — (PDF) [file pone.0145633.s003.pdf]

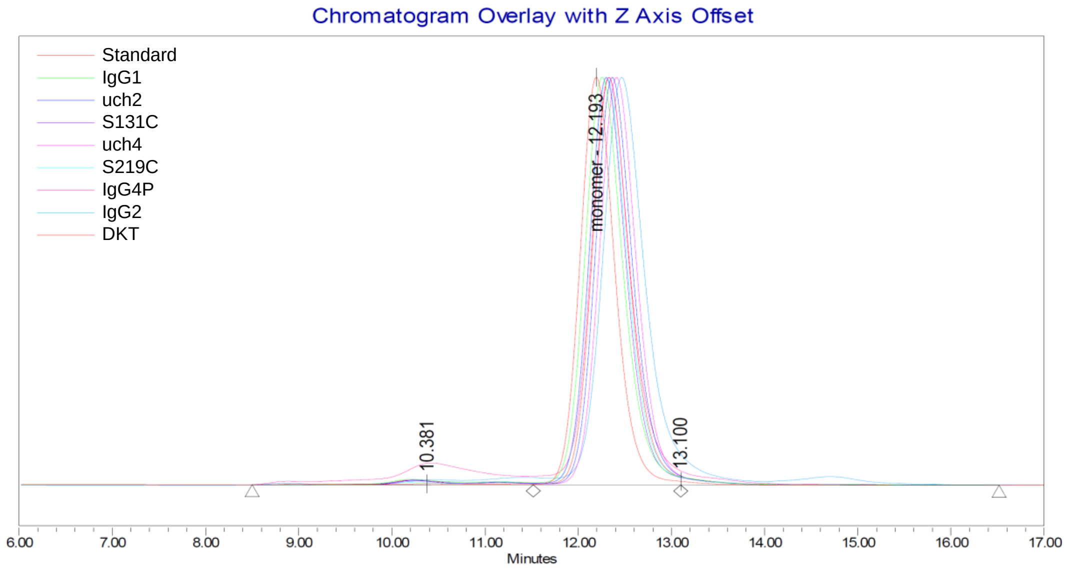

Sample	Monomer (%)	Aggregates (%)	Impurities (%)
Standard	*98.4*	*0.8*	*0.8*
IgG1	95.4	3.0	1.6
uch2	96.3	2.2	1.5
S131C	95.5	2.8	1.6
uch4	96.0	2.4	1.6
S219C	96.4	2.0	1.6
IgG4P	85.6	11.8	2.6
IgG2	91.0	4.5	4.5
DKT	96.1	2.2	1.7

S3 Fig Representative purity analysis of antibody preparations by HP-SEC

Shown are representative high performance size exclusion chromatography (HP-SEC) chromatrogram traces (overlaid) as well as tabular results summarizing monomer, aggregate and impurity content. The average monomer content across all preparations was 94±3%.
