# Supplementary material for: Reformatting Rituximab into Human IgG2 and IgG4 Isotypes Dramatically Improves Apoptosis Induction In Vitro
Source: PLoS One. 2015 Dec 29;10(12):e0145633. doi: 10.1371/journal.pone.0145633 (PMC4694715; doi:10.1371/journal.pone.0145633)
Supplement: S4 Fig — (PDF) [file pone.0145633.s004.pdf]

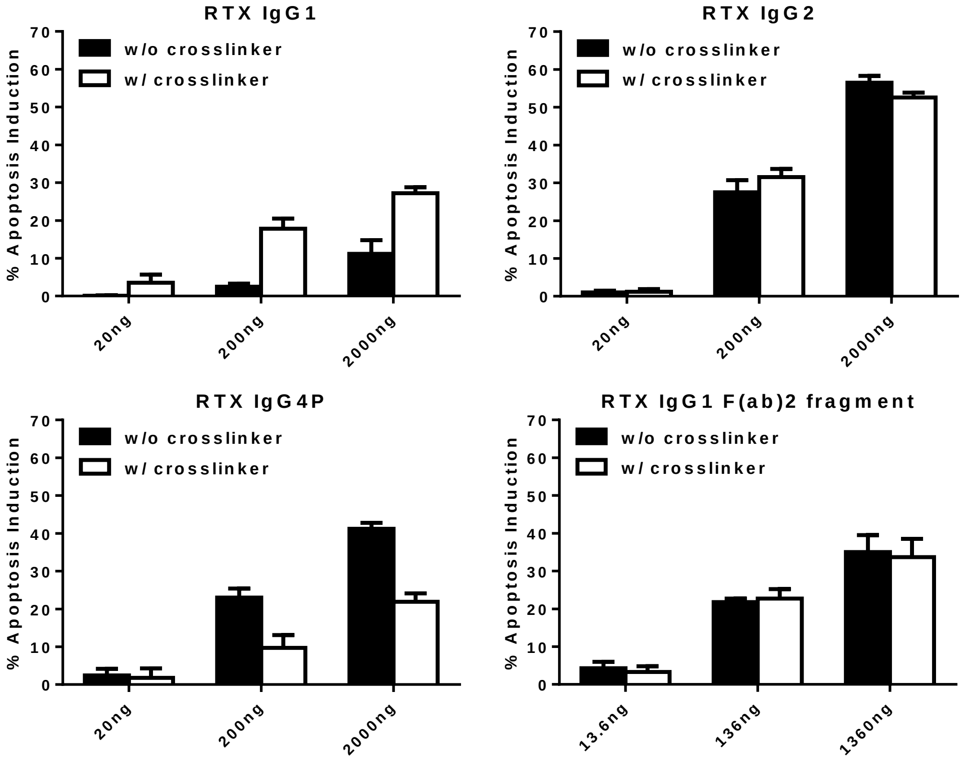

S4 Fig Dose Response analysis of apoptosis induction

Illustrated are the dose dependent abilities of 4 RTX antibody variants to induce apoptosis in Ramos cells. Proteins were used at 20ng, 200ng or 2000ng for full length antibodies and 13.6ng, 136ng and 1360ng for the F(ab)2 fragment. Under the assay conditions (200µl, 100.000 cells per well) this equates to 0.67nM, 6.7nM and 66.7nM respectively. All antibodies show a clear dose response relationship in their ability to induce apoptosis. Shown are the means±standard deviations of three independent assays.
