# Supplementary material for: Reformatting Rituximab into Human IgG2 and IgG4 Isotypes Dramatically Improves Apoptosis Induction In Vitro
Source: PLoS One. 2015 Dec 29;10(12):e0145633. doi: 10.1371/journal.pone.0145633 (PMC4694715; doi:10.1371/journal.pone.0145633)
Supplement: S5 Fig — (PDF) [file pone.0145633.s005.pdf]

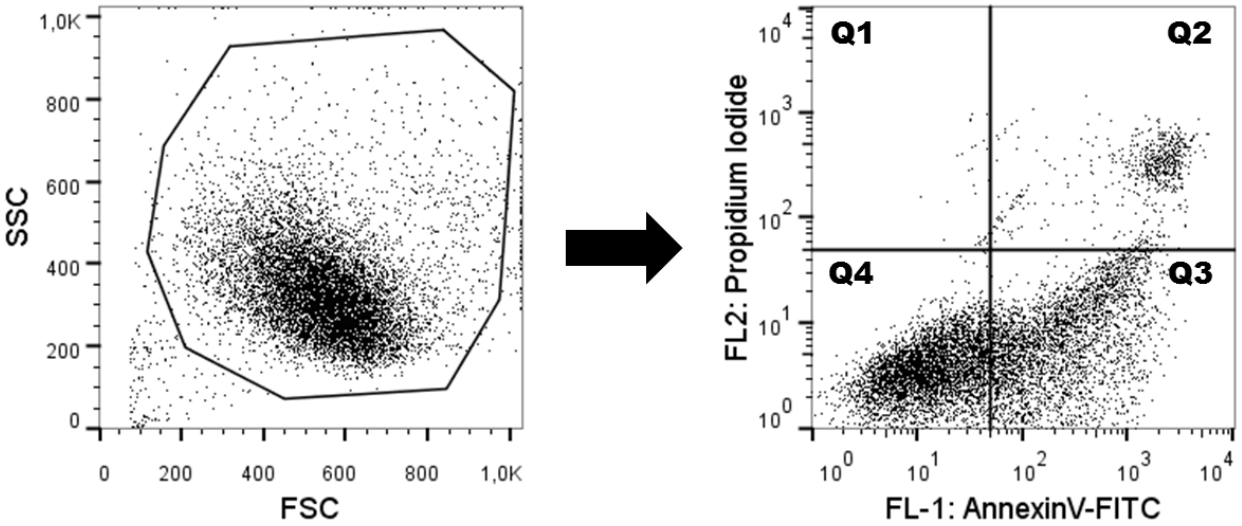

S5 Fig Flow cytometry gating strategy employed to identify apoptotic Ramos cells

The figure illustrates the gating strategies employed in the apoptosis assays. A FSC/SSC dot blot (left) was used to gate the main cell population. This population was then analyzed in a FL-1/FL-2 plot in order to quantify the percentages of AnnexinV-FITC and/or PI positive cells (right). The apoptotic population is found in quadrants (Q) 2 and 3.
