# Supplementary material for: Reformatting Rituximab into Human IgG2 and IgG4 Isotypes Dramatically Improves Apoptosis Induction In Vitro
Source: PLoS One. 2015 Dec 29;10(12):e0145633. doi: 10.1371/journal.pone.0145633 (PMC4694715; doi:10.1371/journal.pone.0145633)
Supplement: S6 Fig — (PDF) [file pone.0145633.s006.pdf]

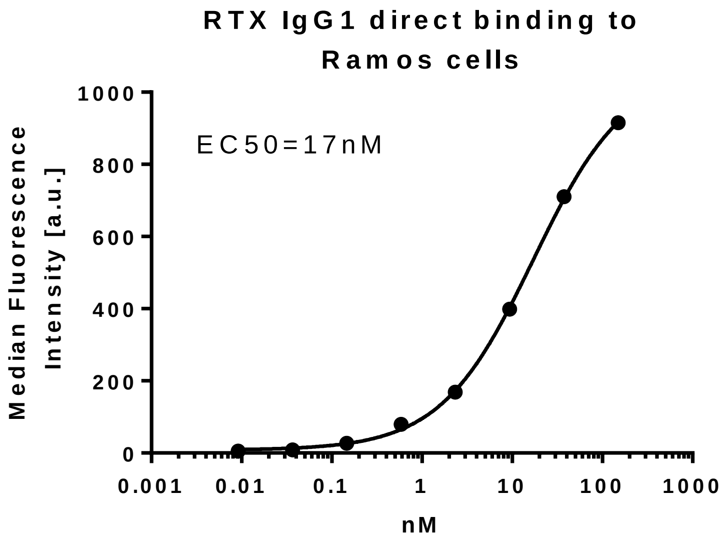

S6 Fig RTX IgG1 saturation binding curve

The figure illustrates the binding of RTX IgG1 to Ramos cells. 1.000.000 cells in 200µl were stained in each sample and bound RTX IgG1 was detected via a polyclonal FITC labeled goat anti human IgG (Jackson Immunoresearch). The EC50 value was determined by fitting the curve using a 4 parametric logistic dose response model. The EC50 of 17nM equates to an absolute amount of 0.5µg of antibody under the employed assay conditions.
