# Supplementary material for: Reformatting Rituximab into Human IgG2 and IgG4 Isotypes Dramatically Improves Apoptosis Induction In Vitro
Source: PLoS One. 2015 Dec 29;10(12):e0145633. doi: 10.1371/journal.pone.0145633 (PMC4694715; doi:10.1371/journal.pone.0145633)
Supplement: S7 Fig — (PDF) [file pone.0145633.s007.pdf]

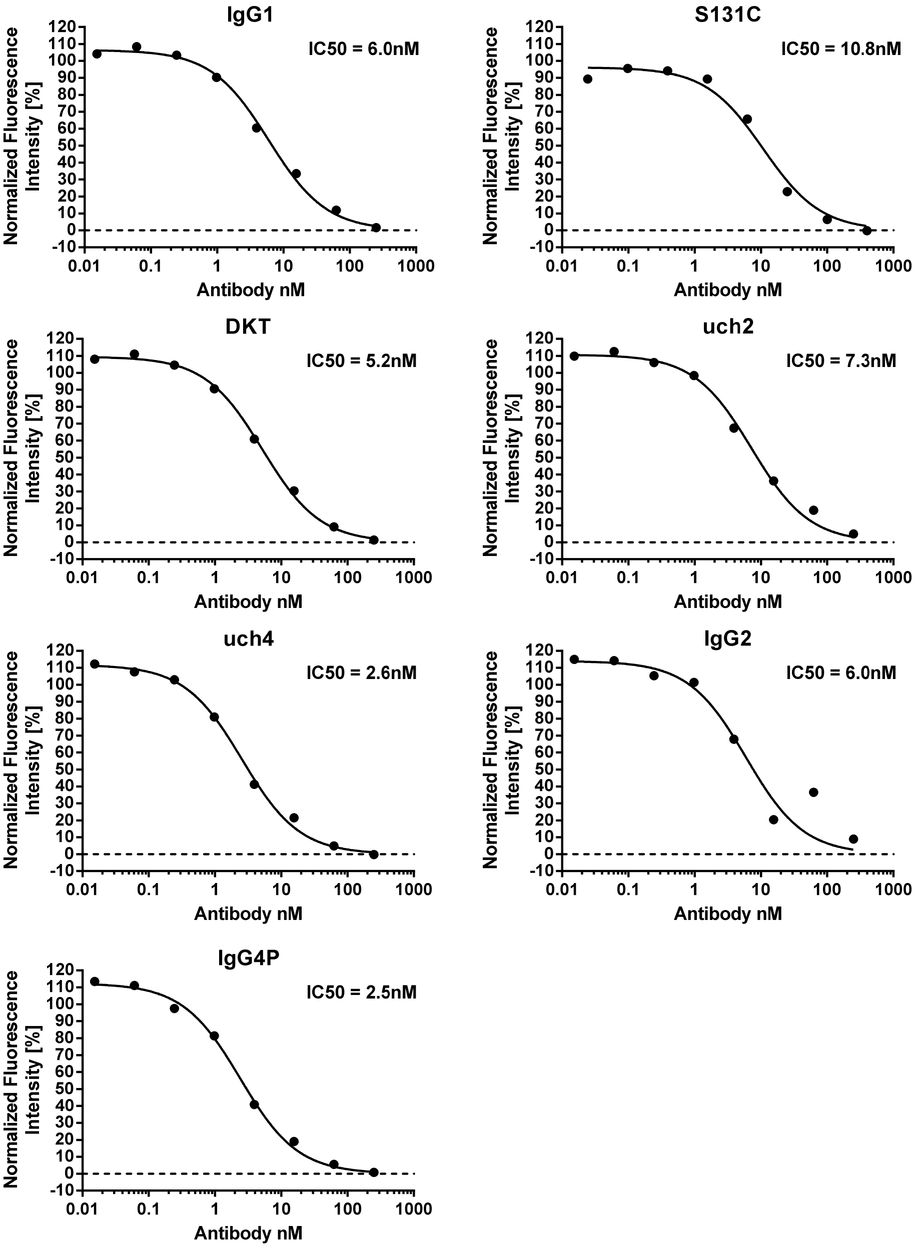

S7 Fig Competition binding assay for RTX variants

Illustrated are flow cytometry competition binding assay for selected RTX antibody variants. Ramos cells were co-incubated with constant amount of FITC labeled anti-CD20 antibody and varying concentrations of the respective RTX variant. The measured fluorescence intensity was normalized to the FITC signal in absence of competing RTX antibody (=100%) and the signal obtained from unstained cells (=0%). Data points were plotted and fitted with a 4 parametric logistic dose response model to obtain IC50 values. All RTX variants present similar curves and comparable IC50 values (mean IC50=5.8±2.8nM) and therefore exhibit comparable affinity to CD20.
