# Supplementary material for: Reformatting Rituximab into Human IgG2 and IgG4 Isotypes Dramatically Improves Apoptosis Induction In Vitro
Source: PLoS One. 2015 Dec 29;10(12):e0145633. doi: 10.1371/journal.pone.0145633 (PMC4694715; doi:10.1371/journal.pone.0145633)
Supplement: S1 Table — (DOCX) [file pone.0145633.s008.docx]

| **Variant** | **Mean Apoptotic Activity %** | **Standard Deviation %** | **N** | **p-value** |
| --- | --- | --- | --- | --- |
| IgG1 a1 | 11 | 3 | 10 | - |
| IgG1 a2 | 10 | 3 | 5 | ns |
| IgG2 | 49 | 6 | 9 | **** |
| IgG4P^#^ | 41 | 8 | 9 | **** |
| IgG4DM^##^ | 36 | 4 | 2 | **** |
| IgG1 a1 F(ab)_2_ fragment | 35 | 5 | 3 | **** |
| IgG2 F(ab)_2_ fragment | 56 | 2 | 3 | **** |
| IgG4P F(ab)_2_ fragment | 45 | 23 | 3 | **** |
| IgG1 a1 Fc fragment | 0.9 | 0.8 | 3 | * |
| negative | 0.2 | 0.4 | 5 | ** |
| ^#^IgG4 subtype variant with hinge mutation S228P  ^##^IgG4 subtype variant with hinge mutations S228P/L234E | | | | |

**S1 Table Absolute apoptotic activity values for different IgG isotypes and fragments (p-values were calculated by one-way ANOVA using the IgG1 a1 as the control group). ns not significant (p>0.05) * p≤0.05; **≤p0.01; *** p≤0.001; **** p≤0.0001**
