# Supplementary material for: Reformatting Rituximab into Human IgG2 and IgG4 Isotypes Dramatically Improves Apoptosis Induction In Vitro
Source: PLoS One. 2015 Dec 29;10(12):e0145633. doi: 10.1371/journal.pone.0145633 (PMC4694715; doi:10.1371/journal.pone.0145633)
Supplement: S2 Table — (DOCX) [file pone.0145633.s009.docx]

|  |  | **without cross-linking mAb** | | | | **with cross-linking mAb** | | |  |
| --- | --- | --- | --- | --- | --- | --- | --- | --- | --- |
| **Mutant ID** | **Type** | **Mean normalized apoptosis %** | **Standard deviation %** | **N** | **p-value** | **Mean normalized apoptosis %** | **Standard deviation %** | **N** | **p-value** |
| IgG1 | control | 100 | 0 | 58 | - | 283 | 110 | 56 | - |
| IgG2 | control | 546 | 155 | 54 | **** | 551 | 171 | 52 | **** |
| IgG4P | control | 441 | 123 | 17 | **** | 260 | 97 | 17 | ns |
| negative | control | 2 | 14 | 47 | **** | 3 | 14 | 47 | **** |
| D | hinge mutant | 116 | 33 | 6 | ns | 198 | 87 | 5 | ns |
| K | hinge mutant | 132 | 24 | 7 | ns | 286 | 112 | 6 | ns |
| T | hinge mutant | 172 | 56 | 7 | ns | 288 | 116 | 6 | ns |
| DT | hinge mutant | 302 | 84 | 4 | ** | 386 | 114 | 4 | ns |
| DK | hinge mutant | 300 | 63 | 4 | ** | 339 | 93 | 4 | ns |
| KT | hinge mutant | 300 | 74 | 4 | ** | 405 | 37 | 4 | ns |
| DKT | hinge mutant | 347 | 99 | 10 | **** | 224 | 98 | 9 | ns |
| DT-S219C | hinge mutant | 603 | 145 | 6 | **** | 783 | 187 | 7 | **** |
| DK-S219C | hinge mutant | 660 | 175 | 6 | **** | 802 | 204 | 6 | **** |
| KT-S219C | hinge mutant | 418 | 143 | 9 | **** | 406 | 152 | 9 | ns |
| DKT-S219C | hinge mutant | 463 | 160 | 8 | **** | 464 | 162 | 8 | ** |
| lh2 | hinge mutant | 129 | 31 | 4 | ns | 292 | 129 | 4 | ns |
| lh4 | hinge mutant | 118 | 49 | 8 | ns | 180 | 53 | 7 | ns |
| uch2 | hinge mutant | 571 | 135 | 4 | **** | 495 | 145 | 4 | * |
| uch4 | hinge mutant | 419 | 68 | 4 | **** | 344 | 134 | 4 | ns |
| P217R | hinge mutant | 138 | 29 | 5 | ns | 293 | 105 | 5 | ns |
| P217S | hinge mutant | 113 | 43 | 5 | ns | 302 | 107 | 5 | ns |
| S219A | hinge mutant | 123 | 31 | 6 | ns | 321 | 100 | 6 | ns |
| S219C | hinge mutant | 530 | 150 | 7 | **** | 586 | 163 | 7 | **** |
| S219D | hinge mutant | 147 | 31 | 12 | ** | 265 | 99 | 11 | ns |
| S219E | hinge mutant | 135 | 33 | 12 | ns | 262 | 80 | 11 | ** |
| S219F | hinge mutant | 318 | 139 | 6 | **** | 441 | 138 | 5 | ns |
| S219G | hinge mutant | 144 | 25 | 7 | ns | 330 | 75 | 6 | ns |
| S219H | hinge mutant | 147 | 21 | 5 | ns | 258 | 82 | 5 | ns |
| S219I | hinge mutant | 136 | 19 | 6 | ns | 279 | 73 | 6 | ns |
| S219K | hinge mutant | 149 | 30 | 11 | ** | 298 | 89 | 11 | ns |
| S219L | hinge mutant | 135 | 26 | 6 | ns | 285 | 66 | 6 | ns |
| S219M | hinge mutant | 145 | 22 | 5 | ns | 286 | 88 | 5 | ns |
| S219N | hinge mutant | 127 | 31 | 11 | ns | 276 | 87 | 12 | ns |
| S219P | hinge mutant | 138 | 35 | 7 | ns | 322 | 82 | 6 | ns |
| S219Q | hinge mutant | 141 | 35 | 10 | ns | 288 | 97 | 11 | ns |
| S219R | hinge mutant | 142 | 40 | 8 | ns | 272 | 79 | 7 | ns |
| S219T | hinge mutant | 127 | 33 | 10 | ns | 254 | 60 | 10 | ns |
| S219V | hinge mutant | 130 | 41 | 3 | ns | 291 | 71 | 3 | ns |
| S219W | hinge mutant | 218 | 34 | 7 | **** | 268 | 72 | 7 | ns |
| S219Y | hinge mutant | 205 | 71 | 6 | **** | 317 | 85 | 5 | ns |
| S219Δ | hinge mutant | 325 | 43 | 7 | **** | 278 | 59 | 7 | ns |
| C220G | hinge mutant | 106 | 30 | 5 | ns | 359 | 93 | 5 | ns |
| S219C-C220G | hinge mutant | 366 | 69 | 5 | **** | 421 | 91 | 5 | ns |
| H224V | hinge mutant | 144 | 25 | 4 | ns | 385 | 150 | 4 | ns |
|  |  | **without cross-linking mAb** | | | | **with cross-linking mAb** | | | |
| **Mutant ID** | **Type** | **Mean normalized apoptosis %** | **Standard deviation %** | **N** | **p-value** | **Mean normalized apoptosis %** | **Standard deviation %** | **N** | **p-value** |
| H224P | hinge mutant | 106 | 17 | 4 | ns | 322 | 107 | 4 | ns |
| T225P | hinge mutant | 142 | 10 | 4 | ns | 350 | 60 | 4 | ns |
| T225E | hinge mutant | 137 | 9 | 4 | ns | 386 | 81 | 4 | ns |
| H224P-T225P | hinge mutant | 88 | 25 | 5 | ns | 349 | 66 | 5 | ns |
| H224V-T225E | hinge mutant | 138 | 18 | 4 | ns | 389 | 65 | 4 | ns |
| lCH1 | CH1 mutant | 229 | 67 | 6 | ** | 335 | 124 | 7 | ns |
| uCH1 | CH1 mutant | 383 | 78 | 6 | **** | 308 | 87 | 6 | ns |
| S131C | CH1 mutant | 334 | 47 | 4 | **** | 367 | 96 | 4 | ns |
| K133R | CH1 mutant | 140 | 36 | 4 | ns | 306 | 93 | 4 | ns |
| S131C-K133R | CH1 mutant | 406 | 61 | 4 | **** | 380 | 77 | 4 | ns |
| G137E-G138S | CH1 mutant | 78 | 16 | 5 | ns | 332 | 126 | 5 | ns |
| S192N | CH1 mutant | 144 | 52 | 6 | ns | 314 | 112 | 6 | ns |
| L193F | CH1 mutant | 149 | 40 | 5 | ns | 356 | 128 | 5 | ns |
| S192N-L193F | CH1 mutant | 146 | 36 | 4 | ns | 331 | 113 | 4 | ns |
| Q196K | CH1 mutant | 131 | 36 | 4 | ns | 324 | 122 | 4 | ns |
| R214T | CH1 mutant | 116 | 13 | 5 | ns | 314 | 88 | 5 | ns |
| uch2-S131C | hinge/CH1 combination | 593 | 206 | 4 | **** | 606 | 166 | 4 | **** |
| uch4-S131C | hinge/CH1 combination | 556 | 189 | 4 | **** | 510 | 116 | 4 | ** |
| R214T-P217R | hinge/CH1 combination | 112 | 38 | 4 | ns | 309 | 154 | 4 | ns |
| R214T-P217S | hinge/CH1 combination | 121 | 39 | 2 | ns | 351 | 139 | 2 | ns |
| DT-S131C | hinge/CH1 combination | 633 | 181 | 8 | **** | 668 | 204 | 8 | **** |
| DT-S131C-S219C | hinge/CH1 combination | 411 | 146 | 8 | **** | 438 | 145 | 8 | * |
| DK-S131C | hinge/CH1 combination | 524 | 137 | 5 | **** | 606 | 193 | 6 | **** |
| DK-S131C-S219C | hinge/CH1 combination | 393 | 168 | 4 | **** | 360 | 112 | 3 | ns |
| KT-S131C | hinge/CH1 combination | 550 | 150 | 8 | **** | 580 | 122 | 6 | **** |
| KT-S131C-S219C | hinge/CH1 combination | 219 | 50 | 9 | * | 267 | 88 | 9 | ns |
| DKT-S131C | hinge/CH1 combination | 473 | 133 | 5 | **** | 425 | 141 | 4 | ns |
| DKT-S131C-S219C | hinge/CH1 combination | 427 | 154 | 9 | **** | 320 | 92 | 9 | ns |
| S131C-S219C | hinge/CH1 combination | 112 | 62 | 9 | ns | 377 | 131 | 9 | ns |

**S2 Normalized apoptotic activity values (p-values were calculated by one-way ANOVA using the IgG1 control without or with crosslinker as the respective control group).**

**ns not significant (p>0.05) * p≤0.05; **≤p0.01; *** p≤0.001; **** p≤0.0001**
